# PacBio Long-Read Sequencing Transcriptome Dataset of Adult *Harmonia axyridis* Under Diapause Inducing and Reproductive Inducing Photoperiod

**DOI:** 10.3389/fgene.2020.01010

**Published:** 2020-09-11

**Authors:** Qiao Gao, Wen Liu, Jia-Lu Wang, Xiao-Ping Wang, Xing-Miao Zhou

**Affiliations:** Hubei Key Laboratory of Insect Resources Utilization and Sustainable Pest Management, College of Plant Science and Technology, Huazhong Agricultural University, Wuhan, China

**Keywords:** *Harmonia axyridis*, long-read sequencing, annotation, long non-coding RNA, alternative splicing

## Introduction

*Harmonia axyridis* is native to Asia, where it is an important biological control agent of aphids and coccids in different ecosystems due to its strong environmental adaptation (Dobzhansky, 1933; Brown et al., 2011; Vandereycken et al., 2012; Riddick, 2017). Introduced to Europe, North and South America and Africa as a biological control agent, it soon became an invasive alien species (Brown et al., 2011, 2018; Roy et al., 2016). These two aspects have attracted considerable research interest, especially with respect to its environmental adaptation and invasion mechanism. Reproductive diapause is an important environmental adaptation strategies for insects to survive the adverse environment (Denlinger, 2008). The diapause stage is a long-term natural developmental arrest stage which have high resistant to a wide range of temperature and other environmental challenges (Denlinger, 2008). Previous studies show that *H. axyridis* enter adult diapause under short-day photoperiod while go for directly development under long-day photoperiod condition (Ongagna and Iperti, 1994; Berkvens et al., 2008; Zhang et al., 2014; Gao et al., 2019). More than 90% of beetles entered diapause when the newly emerged adults reared at 20°C under a short-day (10:14 h light: dark) condition for 20 days, whereas those reared under a long-day (14:10 h light: dark) condition at the same temperature became reproductive (Zhang et al., 2014; Gao et al., 2019). By utilizing the diapause stage of beetle people could get a good way to storage and increase the shelf-life of this biological control agent and obtain a good stage of choice for shipping of commercially viable biological control organisms (Denlinger, 2008; Ruan et al., 2012; Gao et al., 2019). Understand the molecular mechanism of diapause regulation could help people manipulate the diapause of biological control organisms, however this field of *H. axyridis* remains largely blank. In addition, previous studies suggest that the change of diapause characteristics may have helped *H. axyridis* colonize new environments (Raak-van den Berg et al., 2012; Reznik et al., 2015). However, understanding exactly how it was able to invade such a wide range of habitats requires further research on the invasion mechanism. For further molecular mechanism research, although the genome of *H. axyridis* has been assembled (Ando et al., 2018; Gautier et al., 2018), no annotation information is yet available. Therefore, an accurate and well-annotated transcriptome dataset sequencing from beetles under diapause inducing and reproductive inducing photoperiod condition can greatly facilitate research on these topics.

In this study, we used the third-generation long-read sequencing (LRS) method based on the Pacific Biosciences Sequel platform to obtain a full-length transcriptome dataset of *H. axyridis*. The third-generation LRS methods are capable of identifying full-length transcripts, which allows for the direct identification of alternatively transcribed or processed transcripts and polycistronic transcription units (Sharon et al., 2013; Chen et al., 2017; Tombacz et al., 2018). Moreover, the high consensus accuracy of Pacific Biosciences sequencing leads to no systematic errors compared to the second-generation sequencing method (Miyamoto et al., 2014).

To obtain comprehensive transcriptome information on adult *H. axyridis* under reproductive inducing (long-day photoperiod) and diapause inducing (short-day photoperiod) photoperiod, we pooled the total RNA of female and male adults reared under short-day (10:14 h light: dark, 20°C) and long-day (14:10 h light: dark, 20°C) conditions for 2, 4, 8, 12, and 20 days. Three single-molecule real-time (SMRT) cells were sequenced, generating 1,801,619 reads of insert (ROI). This dataset contains 78,379 high-quality consensus isoforms, and all those isoforms were successfully annotated. Coding DNA sequences (CDS) and simple sequence repeats (SSRs) in transcripts were detected, and alternative splicing events, repeat elements, long non-coding RNAs (lncRNAs), and other non-coding RNAs (tRNA, snRNA, snoRAN, rRNA) were predicted. The raw reads, specific techniques and experimental details used to obtain our datasets are publicly available to enable the reanalysis of our raw read data with different methods and for our datasets to be used as references for other RNA-based studies of *H. axyridis*. Because we analyzed pooled samples from different ages, sexes, and diapause and reproductive inducing photoperiods, the transcriptome information we obtained can provide a basis for research on the molecular mechanisms underlying developmental regulation or diapause adaptation.

## Value of Data

- Although the genome of *H. axyridis* has been assembled, no annotation information is yet available. Therefore, our study provided an accurate and well-annotated transcriptome dataset sequencing from beetles under diapause inducing and reproductive inducing photoperiod condition, detected the Coding DNA sequence and simple sequence repeats in transcripts and predicted the alternative splicing events and long non-coding RNAs (lncRNAs), which could greatly facilitate research on the relative topics.
- The raw reads, specific techniques, and experimental details used to obtain our datasets are publicly available to enable the reanalysis of our raw read data with different methods and for our datasets to be used as references for other RNA-based studies of *H. axyridis*.

## Data

**Figure 1:**
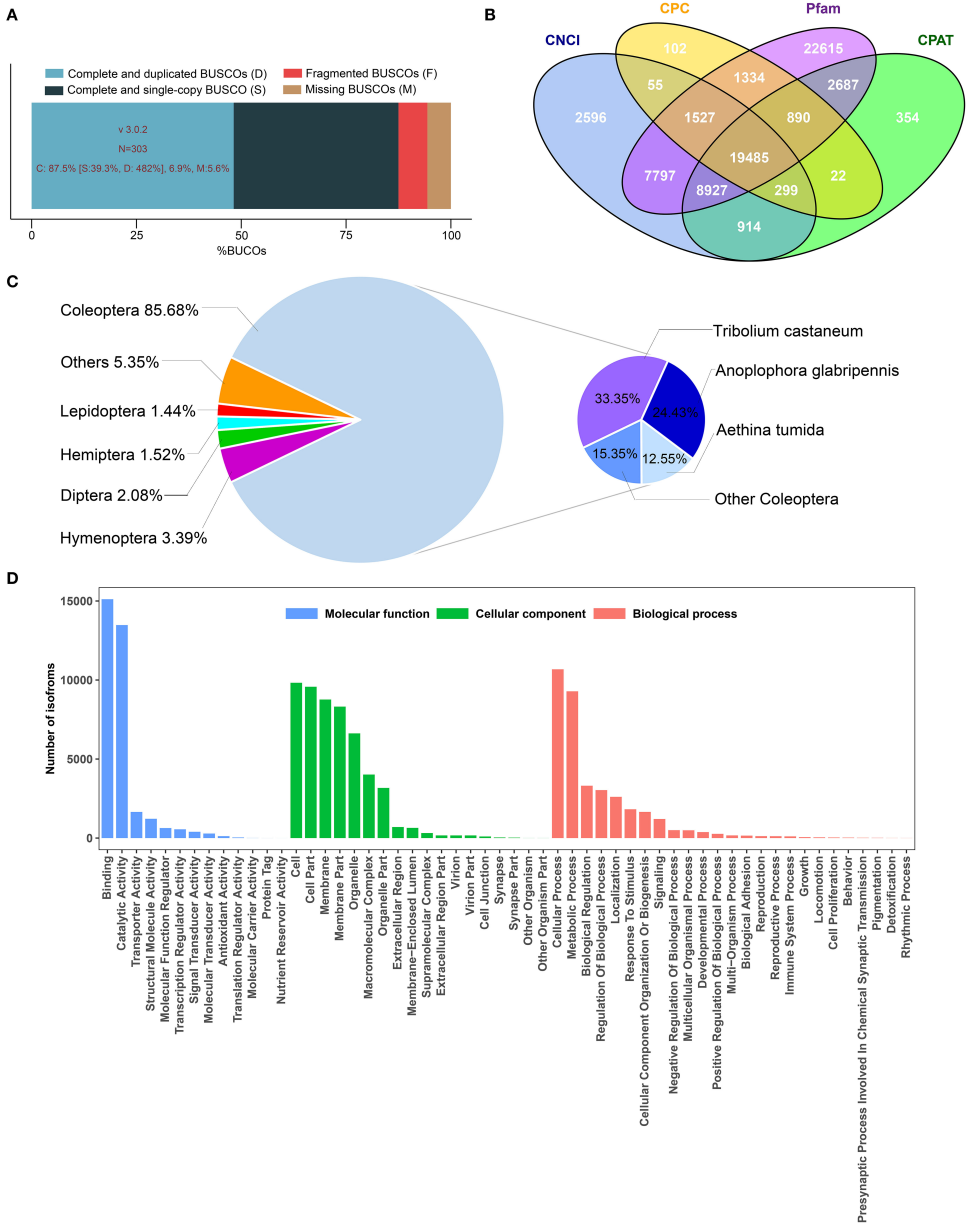
Summary of transcriptome results obtained from PacBio sequencing platforms. **(A)** BUSCO analysis show proportions classified as complete (C, blues), complete duplicated (D, light blue), complete single-copy (S, dark blue), fragmented (F, red), and missing (M, brown). **(B)** Long non-coding RNAs were predicted by Calculator (CPC), Coding-Non-coding Index (CNCI), Coding Potential Assessment Tool (CPAT), and Pfam protein structure domain analysis. **(C)** Homologous species distribution of *H. axyridis* annotated in the NR database. **(D)** GO classification show transcripts were classified into three main categories: Molecular function, cellular component, and biological process.

In this study, the full-length transcriptome dataset of *H. axyridis* was generated with a pooled sample based on the Pacific Biosciences Sequel platform. Three non-size-selected libraries were generated, including 1,879,165 polymerase reads. After removing the adaptor, we obtained 17,126,082 subreads. Subreads belonging to the same polymerase read were clustered and polished to one single ROI. On the basis of the conditions of full passes of ≥0 and quality of >0.75, 1,801,619 ROIs were obtained with a mean length of 2,296 bp (Table 1). The sequencing quality of ROIs were show in Supplementary Figures 1–3. After the SMRT analysis, 155,220 high-quality consensus isoforms and 125,470 low-quality consensus isoforms were obtained (SRP174447). The high-quality consensus isoforms of each library were merged to yield the final results, and redundancy was removed. Finally, 78,379 final high-quality consensus isoforms were generated with a total base of 105,960,416 bp, a mean length of 1,352 bp and an N50 of 1,802 bp. The GMAP analysis shows that 88.2% (69,132/78,379) of the redundancy-removed transcripts could be mapped to the reference genome. BUSCO completeness analysis showed that 87.5% of BUSCOs were complete (Figure 1A).

**Table 1:** Data statistics of reads of insert.

**Library**	**Library size (kbp)**	**Reads of insert**	**Mean quality**	**Mean length (bp)**	**Mean passes**
r54270_20180519_103727-1_D01	0–10	587,563	0.89	2,261	10
r54266_20180522_112710-1_E01	0–10	572,303	0.88	2,348	7
r54267_20180522_112541-1_F01	0–10	641,753	0.87	2,278	6
Total	/	1,801,619	0.88	2,296	7.67

High-quality consensus isoforms were annotated with public databases, including NT, NR, GO, KOG, KEGG, Swiss-Prot, and InterPro (Table 2). All the isoforms were annotated and most transcripts were annotated in NR (77.09%), KEGG (63.91%), and Swiss-Prot (61.66%). The homologous species distribution of *H. axyridis* annotated in the NR database indicated that the *H. axyridis* sequences show high homology with *Tribolium castaneum* (33.3%), *Anoplophora glabripennis* (24.2%), and *Aethina tumida* (22.5%) sequences (Figure 1C). GO classification revealed that 29,961 isoforms corresponded to 56 specific GO terms (Figure 1D).

**Table 2:** Annotation statistics.

**Database**	**Number**	**Percentage**
NR	60,421	77.09%
NT	23,093	29.46%
Swissprot	48,325	61.66%
KEGG	50,090	63.91%
KOG	46,559	59.40%
Interpro	45,462	58.00%
GO	29,961	38.23%
Intersection	10,270	13.10%
Overall	63,223	80.66%
Total isoforms	78,379	100%

In total, 53,825 CDSs were detected, including 24,921 complete ORFs. A total of 10,975 SSRs were identified, which contained 8,039 mononucleotides, 799 dinucleotides, 2,016 trinucleotides, 51 tetranucleotides, 62 pentanucleotides, and 8 hexanucleotides. A total of 1,163 alternative splicing events were identified. However, because the sequence-level annotation of the reference genome of *H. axyridis* is unavailable, we could not classify the types of alternative splicing events. 20.87% of the high-quality consensus sequence were annotated as repeat elements including 0.79% of simple repeats and 20.08% of unclassified repeats. In total, 19,485 lncRNA transcripts were predicted by all four methods, including CPC, CNCI, CPAT, and Pfam protein structure domain analysis (Figure 1B). And 6,654 other non-coding RNAs were predicted including 143 tRNA, 261 snRNA, 766 snoRAN, and 5,486 rRNA. The annotation information, detected SSR, predicted alternative splicing, repeat elements and non-coding RNAs were deposited in Figshare (https://doi.org/10.6084/m9.figshare.8131775).

## Materials and Methods

### Sample Collection

*Harmonia axyridis* specimens of the Red-nSpots color pattern (Gautier et al., 2018) were collected in Wuhan, China (30°28′N 114°21′E). The offspring of these insects were reared in cages (40 × 40 × 40 cm) at 25°C under a 14:10 h light:dark photoperiod. Two hundred larvae were reared in one cage and fed on a sufficient number of *Acyrthosiphon pisum* Harris. After pupation, the insects were collected in petri dishes (diameter 9 cm) and randomly assigned to two different photoperiod treatments: a long-day (LD) 14:10 h light:dark photoperiod and a short-day (SD) 10:14 h light:dark photoperiod. The insects in both treatments were kept at 20°C. For each photoperiod treatment, female and male adults were identified and segregated immediately after eclosion. The adults were reared in transparent round boxes (diameter 10 cm, height 5 cm, 20 individuals per box) under the photoperiod treatment condition and fed on a sufficient number of *Acyrthosiphon pisum* Harris. Random samples of five adults of each sex from each treatment group were collected 2, 4, 8, 12, and 20 days after eclosion. These insects were quickly frozen in liquid nitrogen and stored at −80°C for RNA extraction.

### RNA Extraction

Total RNA was extracted from the total insect of each sample using TRIzol reagent (Invitrogen, USA) according to the manufacturer's instructions. RNA concentration was tested with a NanoDrop spectrophotometer (Thermo Fisher Scientific, USA) and its integrity with an Agilent 2100 (Agilent Technologies, USA). Equal amounts of RNA from each sample were pooled for sequencing. To address potential RNA contaminants, rRNA was depleted with the Ribo-Zero Magnetic Kit (Bacteria, Epicenter). The sample was purified with RNAClean XP Beads (Agencourt).

### Sequencing and Library Construction

Sequencing was performed according to the Pacific Biosciences ISO-Seq protocol. Total RNA was used to synthesize cDNA using a Clontech SMARTer PCR cDNA Synthesis Kit. After large-scale PCR, the cDNA was ready for SMRTbell template preparation and sequencing. We pooled a BluePippin size-selected library with a non-size-selected library before sequencing and set the minimum threshold size to >4 kb.

According to the SMRT analysis protocol (SMRT Link v5.1.0.), raw polymerase reads that had full passes ≥0 and predicted consensus accuracy >0.75 were selected for producing ROIs. After detecting poly-A sequences, 5′ adaptors and 3′ adaptors, the ROIs were classified into four categories: full-length non-chimeric, chimeric, non-full-length, and short reads. Only reads with two adaptors and a poly-A tail were classified as full-length. Consensus isoforms were predicted using an iterative clustering (ICE) algorithm 18 for error correction, and the full-length consensus sequences were polished using Quiver to obtain high-quality consensus isoforms for further analysis. Redundancy in high-quality full-length transcripts was removed using CD-HIT-EST based on sequence similarity (Li and Godzik, 2006).

To assess the quality of the transcriptome, the redundancy-removed high-quality full-length transcripts were mapped to the genome sequence of the Red-nSpots form (HaxR) *H. axyridis* (http://bipaa.genouest.org/sp/harmonia_axyridis/download/genome/HaxR_v1.0/) using Genomic Mapping and Alignment (GMAP parameters: allow-close-indels0–min-trimmed-coverage = 0.85–min-identity = 0.90–cross-species) (Wu and Watanabe, 2005; Gautier et al., 2018). The completeness of the transcriptome was assessed using BUSCO v3.0.2.

### Dataset Annotation

Non-redundant transcript sequences were annotated with public databases. Blast v2.2.23 was used to annotate transcripts by aligning them with the Nucleotide sequence database (NT), Non-redundant protein sequence database (NR), EuKaryotic Orthologous Groups (KOG), Kyoto Encyclopedia of Genes and Genomes (KEGG), and Swiss-Prot (Altschul et al., 1990). Blast2GO v2.5.0 was used with NR annotation to perform GO (Gene Ontology) annotation (Conesa et al., 2005). InterProScan 5 5.11-51.0 was used to annotate genes based on InterPro (Quevillon et al., 2005). All software was set to the default parameters.

### CDS Prediction and SSR Detection

TransDecoder v3.0.1 was used to identify CDS. The longest open reading frame (ORF) was extracted, and Blast v2.2.23 was used to search Pfam protein homologous sequences in Swiss-Prot and Hmmscan to predict coding regions. All software was set to the default parameters. MISA v1.024 (parameters: 1–12, 2–6, 3–5, 4–5, 5–4, 6–4, 100, 150) was used to find SSRs in transcripts.

### Alternative Splicing Detection

Alternative splicing events were predicted based on the redundancy-removed transcripts. First, all sequences were aligned to each other with BLAST (Altschul et al., 1997). Then, the alignment results which met the following conditions were considered as alternative splicing events: (1) both sequence lengths were more than 1,000 bp and two high-scoring segment pairs were present in the alignment; (2) the alternative splicing gap was >100 bp with at least 100 bp distance to the 3′/5′ end; and (3) all alternatively spliced transcripts allowed 5 bp overlap (Liu et al., 2017).

### Repeat Elements Prediction

Repeat elements were predicted by using RepeatModeler v1.0.11 and RepeatMasker v4.0.7 (Lerat, 2010). First, all sequences were cut into contigs and built a reference repeat elements library using RepeatModeler. Then RECON and RepeatScout were used to do *de novo* prediction of repeat elements and Repbase were used for classification. Finally, the repeat elements were annotated by RepeatMasker.

### Non-coding RNA Prediction

LncRNAs were predicted by screening the coding potential of transcripts using the most widely used methods: Coding Potential Calculator (CPC) (Kong et al., 2007), Coding-Non-coding Index (CNCI) (Sun et al., 2013), Coding Potential Assessment Tool (CPAT) (Finn et al., 2014), and Pfam protein structure domain analysis (Wang et al., 2013). tRNA, snRNA, snoRAN, and rRNA were predicted by blast the sequence to Rfam database (Rfam v12.3) using Blastall v2.2.24 (Burge et al., 2013).

## Data Availability Statement

The datasets presented in this study can be found in online repositories. The names of the repository/repositories and accession number(s) can be found at: https://www.ncbi.nlm.nih.gov/genbank/, GHLY00000000; https://www.ncbi.nlm.nih.gov/, SRP174447; https://doi.org/10.6084/m9.figshare.8131775.

## Author Contributions

X-PW and X-MZ designed the study. QG, J-LW, and WL carried out the experiments and analyzed the data. QG, WL, and X-PW wrote the paper. All authors contributed to the article and approved the submitted version.

## Conflict of Interest

The authors declare that the research was conducted in the absence of any commercial or financial relationships that could be construed as a potential conflict of interest.

## Supplementary Material

Supplementary Figure 1 Sequencing quality of Reads of Insert in Library r54270_20180519_103727-1_D01.

Supplementary Figure 2 Sequencing quality of Reads of Insert in Library r54266_20180522_112710-1_E01.

Supplementary Figure 3 Sequencing quality of Reads of Insert in Library r54267_20180522_112541-1_F01.
