# Supplementary figures and images for: PacBio Long-Read Sequencing Transcriptome Dataset of Adult Harmonia axyridis Under Diapause Inducing and Reproductive Inducing Photoperiod
Source: Front Genet. 2020 Sep 11;11:1010. doi: 10.3389/fgene.2020.01010 (PMC7518098; doi:10.3389/fgene.2020.01010)

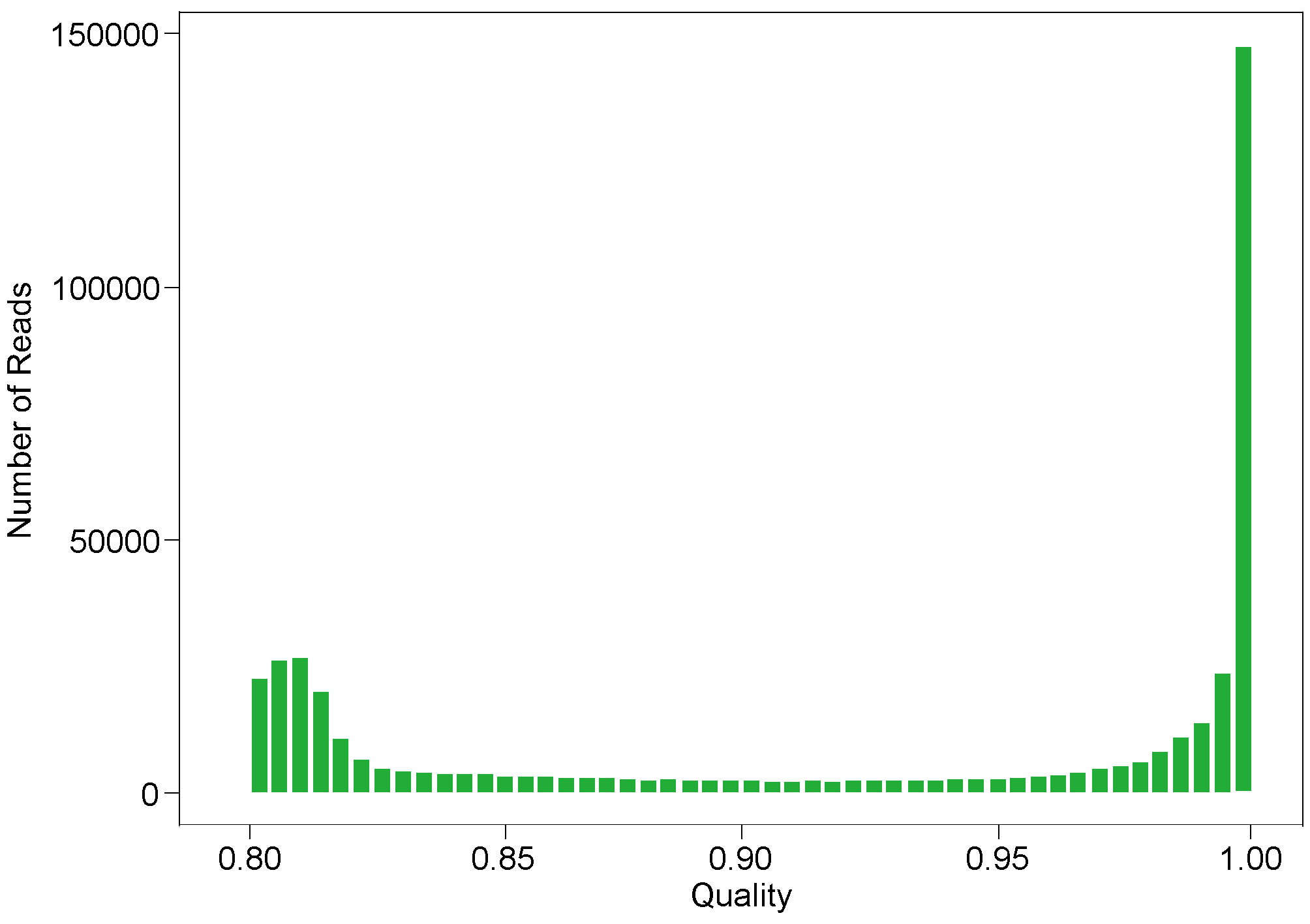

Supplement: Supplementary Figure 1 — Sequencing quality of Reads of Insert in Library r54270_20180519_103727-1_D01. [file Image_1.TIF]

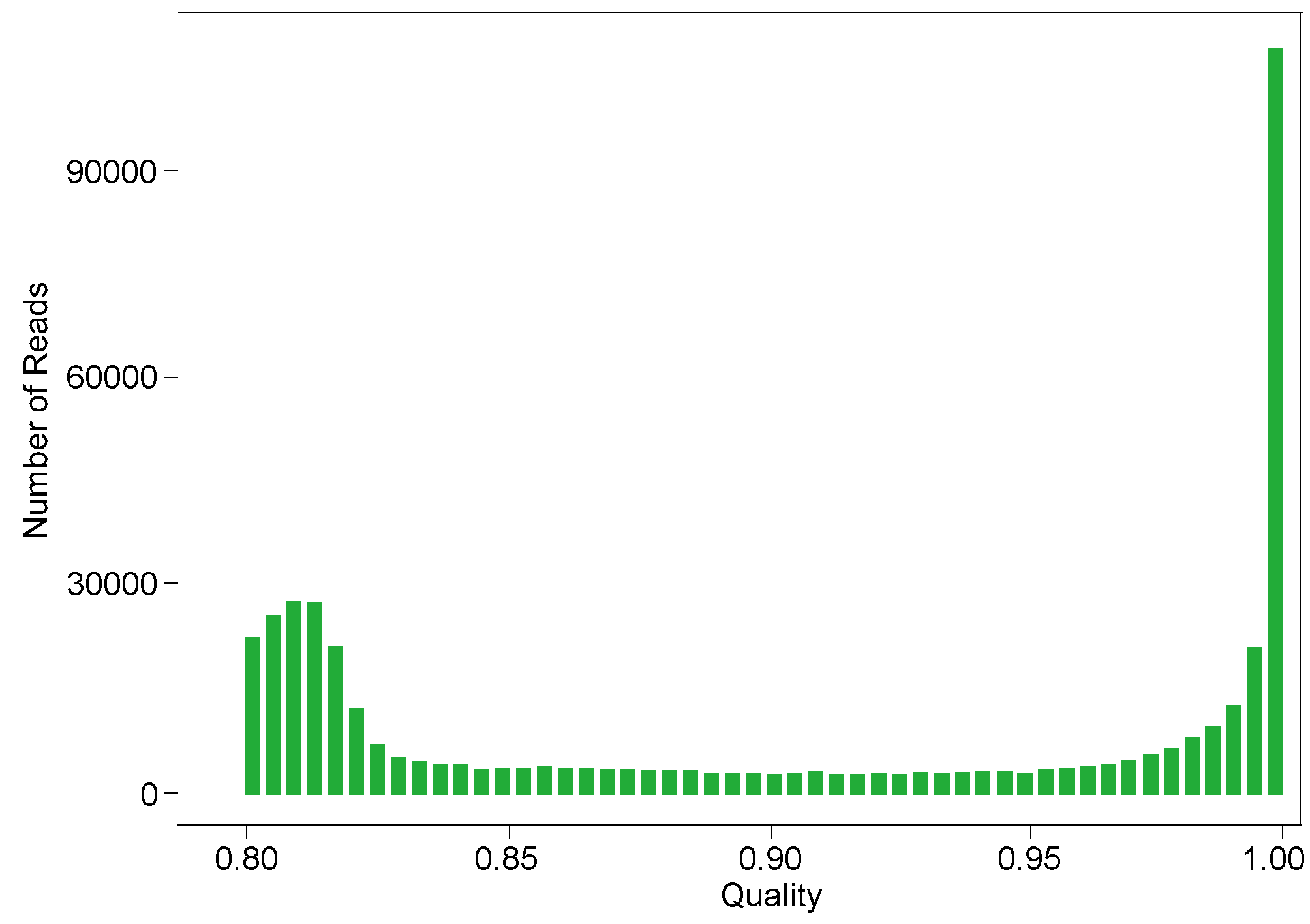

Supplement: Supplementary Figure 2 — Sequencing quality of Reads of Insert in Library r54266_20180522_112710-1_E01. [file Image_2.TIF]

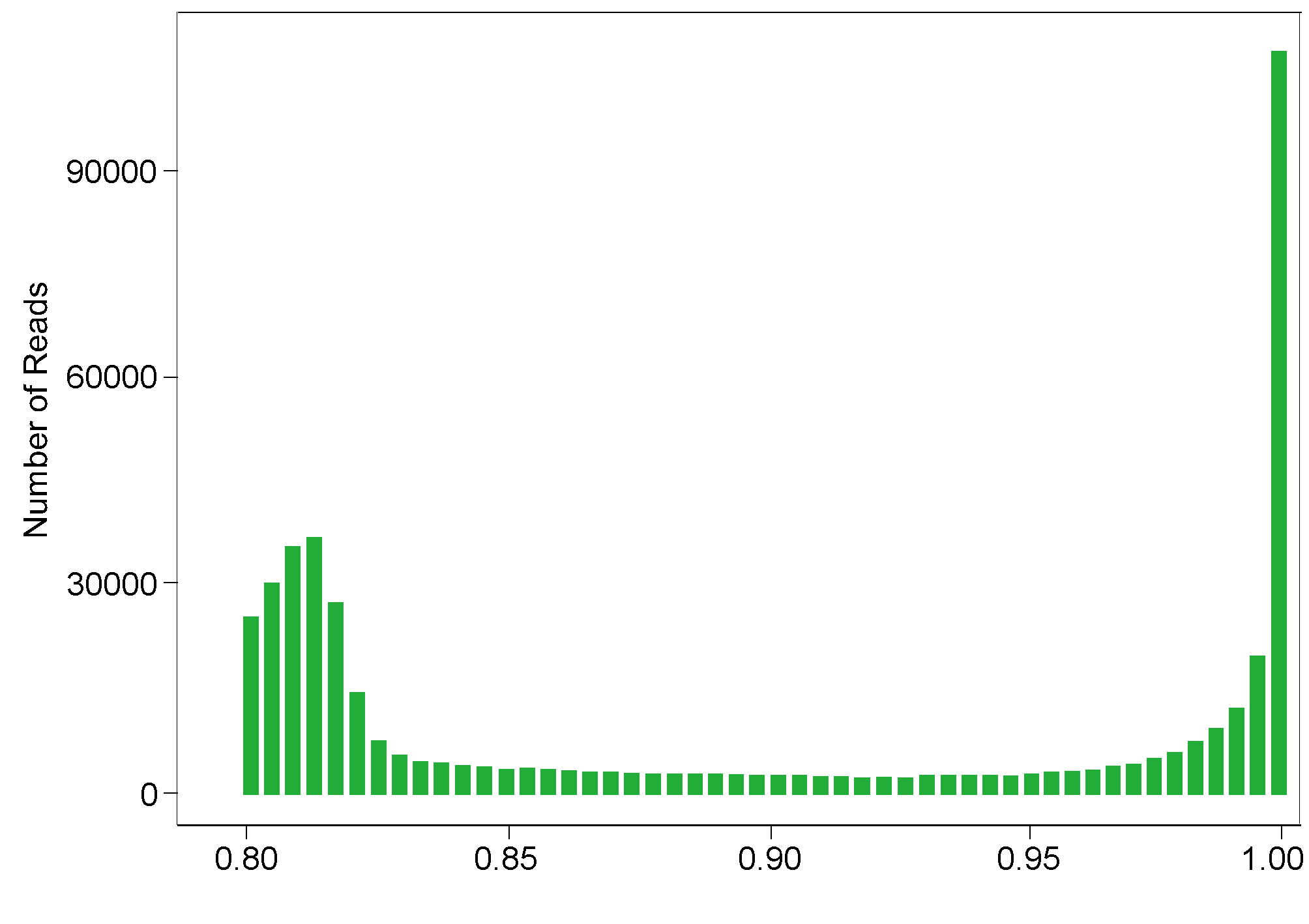

Supplement: Supplementary Figure 3 — Sequencing quality of Reads of Insert in Library r54267_20180522_112541-1_F01. [file Image_3.TIF]
